# Metric Prescriptions

**Published:** 1894-10

**Authors:** A. L. Benedict


### Therapeutic Notes.

### METRIC PRESCRIPTIONS.

(ADAPTED FROM VARIOUS SOURCES.)

BY A. L. BENEDICT, M. D.

#### SUB-ACUTE CYSTITIS, WITH ALKALINE URINE.

| | |
|---|---|
| ℞.—Ext. hyoscyami, fl. | 4.00 |
| Sodii benzoatis | 30.00 |
| Syr. sars. co. | 30.00 |
| Aquam ad. | 100.00 |

S. Shake. 5 c. c. A. C. (Teaspoonful before eating.)

#### ANTIEMETIC IN PERITONITIS, ETC.

| | |
|---|---|
| ℞.—Chloralis | 1.00 |
| Vini ipecacuanhæ | 1.00 |
| Aquam ad. | 100.00 |

S. 5 c. c., every five minutes, p. r. n.

### DIABETES.

| | |
|---|---|
| ℞.—Codeinæ | .03 |
| Ext. ergotæ | .05 |
| Ext. belladonnæ | .01 |

S. In pill, t. i. d.

---

### CHOLAGOGUE.

| | |
|---|---|
| ℞.—Ammonii chlor | 15.00 |
| Ac. hydrochlor., dil | 20.00 |
| Syr. limonis | 20.00 |
| Aquam ad | 100.00 |

S. 5 c. c. P. C. in hepatic torpor.

---

### INFANTILE CONVULSIONS.

| | |
|---|---|
| ℞.—Chloralis | .05 |
| Potassii brom | .25 |
| Mucil. acaciæ ad | 15.00 |

S. Use as enema. Repeat in half to one hour, if necessary.

---

### BLEEDING HEMORRHOIDS.

| | |
|---|---|
| ℞.—Ung. gallæ. | |
| Ung. hydrarg. ammon | āā 15.00 |

---

### MODIFIED DOBELL'S SOLUTION.

| | |
|---|---|
| ℞.—Sodii bicarb | 10.00 |
| Sodii (quadre) boratis | 10.00 |
| Potassii chloratis | 25.00 |
| Ac. carbolici | 2.50 |
| Glycerini | 50.00 |
| Aquam ad | 100.00 |

S. Dilute to 500 c. c. (about one pint). Look out for effervescence.

---

### ALCOHOLISM.

(From prescription written by a patient who was himself a physician.)

| | |
|---|---|
| ℞.—Sodii brom | 20.00 |
| Tr. capsici | 15.00 |
| Spts. ammoniæ arom | 30.00 |
| Tr. belladonnæ | 2.00 |
| Aquam ad | 150.00 |

S. 15 c. c. (tablespoonful), p. r. n. Repeat, with caution.

